# A Phase IB Study of Binimetinib and Palbociclib in Molecularly Selected Advanced Triple-Negative Breast Cancer

**DOI:** 10.1158/2767-9764.CRC-25-0428

**Published:** 2025-09-29

**Authors:** Luis Manso, Rodrigo Sánchez-Bayona, Juan Antonio Guerra, Alfonso Cortés-Salgado, Juan Miguel Cejalvo, José A. García-Saenz, Serafín Morales, Lucía González-Cortijo, Silvana Mourón, María J. Bueno, Leonardo D. Garma, Miguel Quintela-Fandino

**Affiliations:** 1 Medical Oncology Department, Hospital Universitario 12 de Octubre, Madrid, Spain.; 2 Medical Oncology Department, Hospital Universitario de Fuenlabrada, Fuenlabrada, Spain.; 3 Medical Oncology Department, Hospital Universitario Ramón y Cajal, Madrid, Spain.; 4 Medical Oncology Department, Hospital Clinico Universitario, INCLIVA, Medicine Department, Universidad de Valencia, Valencia, Spain.; 5 Medical Oncology Department, Hospital Universitario Clinico San Carlos, Madrid, Spain.; 6 Medical Oncology Department, Hospital Universitario Arnau de Vilanova, Lleida, Spain.; 7 Medical Oncology Department, Hospital Universitario Quironsalud Madrid, Madrid, Spain.; 8 Breast Cancer Clinical Research Unit, Clinical Research Program, CNIO – Spanish National Cancer Research Center, Madrid, Spain.

## Abstract

**Purpose:**

Advanced, pretreated triple-negative breast cancer (TNBC) has a dismal prognosis and lacks effective options beyond standard cytotoxics. We previously showed, via phosphoproteomic screening, that cyclin-dependent kinase 6 (CDK6) and ERK hyperactivation are linked to adverse outcomes and represent actionable targets. This prompted us to evaluate palbociclib and binimetinib in advanced TNBC after one or two prior therapies.

**Patients and Methods:**

Patients with increased ERK and/or CDK6 activity were eligible. Treatment consisted in daily binimetinib (45 mg twice a day) plus palbociclib (100 mg daily, days 1–21) in 28-day cycles. Palbociclib escalation to 125 mg was allowed in cycle 2. The primary objective was to demonstrate a 2.5 month–long progression-free survival (PFS), a 50% increase over the reference PFS for cytotoxics (1.7 months). Whole-exome sequencing was performed in tumor samples of the efficacy population.

**Results:**

Fifty-one patients were screened, of whom 50 were biomarker-positive. Twenty-four initiated treatment between June 2021 and July 2022. Toxicity was frequent, consisting mainly in fatigue, diarrhea, neutropenia, and ocular effects, requiring frequent dose interruptions or reductions. The primary objective was not met (median PFS 50 days). However, a bimodal PFS pattern emerged, with 13% of the patients achieving disease control lasting 4 to 13 months. Whole-exome sequencing revealed a distinct mutational landscape among long-term responders compared with early progressors.

**Conclusions:**

In this biomarker-enriched TNBC population, the combination of palbociclib and binimetinib showed limited activity and notable toxicity. Whereas CDK6 and ERK hyperactivation confirmed their prognostic role, they did not predict treatment benefit. Exploratory genomic findings suggest the existence of a biologically distinct subset of patients with prolonged benefit, encouraging further investigation.

**Significance:**

Previous studies showed that patients with early TNBC with increased CDK4/6 and ERK activity are at high risk of relapse. Preclinical data also suggest the benefit of combined inhibition of CDK4/6 and MEK, and novel therapies are needed for TNBC in the advanced disease setting. Despite the rationale and a biomarker-driven design, this combination was toxic and showed limited efficacy. This combination should not be further developed in this disease.

## Introduction

Metastatic triple-negative breast cancer (TNBC) is almost invariably lethal, with a median overall survival (OS) currently in the range of 24 months since diagnosis (1). Novel therapeutic developments like immune checkpoint inhibition with pembrolizumab (1) or antibody–drug conjugates like sacituzumab govitecan (2) or trastuzumab deruxtecan (3) have improved previously disappointing median OS times in the range of 10 months (4). Targeted agents like PARP inhibitors have improved as well the prognostic landscape for the BRCA1/2-mutant subgroup (5, 6). Nevertheless, although the former agents have approximately doubled the median life expectancy for this disease in the last decade, these achievements are insufficient given the usual clinical characteristics of patients with TNBC: women in their 4th to 5th decade otherwise healthy. Similarly, these new drugs, although in some aspects better tolerated than traditional chemotherapeutic agents, are certainly not devoid of toxicity and have significant quality-of-life limiting effects (7–9). Novel drugs or drug combinations, along with precision oncology biomarkers, are clearly needed to improve this situation.

TNBC displays a highly heterogeneous genomic landscape (10–12). In the past, we observed that diverse genomic profiles converged into distinct aberrant kinase-activation patterns, building a kinome-based classification of this disease (13). This classification had two main patterns: one (30% of the patients) with 0 of 6 hyperactive kinases [P70S6K, ERK1/2, c-KIT, cyclin-dependent kinase 6 (CDK6), PRKCE, and PNKP], and a second one in which tumors displayed one or more (of the 6) hyperactive kinases. The presence of one or more hyperactive kinases in the tumor was linked to a >9-fold higher risk of relapse of early TNBC. More importantly, beyond this prognostic impact, preclinical therapeutic assays demonstrated that targeting the former kinases in pairs was a powerful approach. Dual ERK1/2 and CDK6 targeting demonstrated the most powerful and broadest preclinical effects: besides >99% cytotoxic efficacy in colony assays, the combination of palbociclib and binimetinib extended mice OS up to 5-fold across different models (grafted human and murine TNBC cell lines and one TNBC patient-derived xenograft; ref. 13). Previous preclinical work has shown that MAPK pathway inhibition activates apoptosis but not cell-cycle arrest pathways in cancer cells, and CDK4 activity has been associated with this escape pathway in response to MAPK inhibition. The combination of MEK and CDK4 inhibition has shown promising activity in colorectal cancer and RAF/MEK inhibitor–sensitive and -resistant melanoma models (14–17). Moreover, the combination of palbociclib and binimetinib (targeting CDK4/6 and MEK, respectively) was shown to induce tumor cell senescence and matrix remodeling leading to enhanced drug delivery and T-cell infiltration, supporting further study of the combination.

Motivated by the previous findings, we conducted a phase IB clinical trial in patients with advanced TNBC that showed hyperactivation of CDK6 and/or MEK in their tumor samples, aiming to study a potentially powerful, orally available drug combination and biomarkers for this disease. Nearly all screened patients met the biomarker selection criteria, consistent with our previous observation that CDK6 and/or MEK hyperactivation are strongly enriched in the metastatic TNBC population. Although the study did not meet its primary endpoint, a number of patients experienced a long-lasting benefit from the combination; a distinct mutational profile distinguished these patients from early progressors.

## Materials and Methods

We performed a prospective, open-label, multicentric, single-arm, phase IB investigator-initiated study. The study was conducted in accordance with the Declaration of Helsinki and Good Clinical Practice standards and registered at ClinicalTrials.gov (NCT04494958). Ethics approval was obtained from the Hospital 12 de Octubre Ethics Committee and the Spanish Agency for Medicine and Health Products (code 20/394).

### Study population

Women ≥18 years old were eligible if they had a noncurable, locally advanced or metastatic TNBC histologically confirmed breast cancer. Inclusion criteria were the following: reception of a minimum of one and a maximum of two treatment lines for advanced disease; in case of confirmed BRCA1/2 mutation, at least one treatment line must have contained cisplatin/carboplatin and/or a PARP inhibitor; demonstration of hyperactivation of CDK6 and/or ERK in tumor tissue; confirmed RECIST v1.1 disease progression to the previous treatment line; Eastern Cooperative Oncology Group performance status of 0/1; life expectancy >24 weeks; recovery of residual toxicities to grade 1 or 0; and adequate organic function according to usual criteria. Negative pregnancy test plus adequate contraception measures were mandatory for women with childbearing potential. Central nervous system metastases were not an exclusion criterion if clinically stable for at least a month in the absence of corticosteroid treatment. Other relevant exclusion criteria included the following: concurrent use of strong CYP3A4 inhibitors/inducers; history of QT syndrome, Brugada syndrome, QTc prolongation, or Torsade de Pointes; Gilbert syndrome; presence of neuromuscular disorders associated with elevated creatine kinase; malabsorption or other conditions that could interfere with the absorption of oral study medication; and current diagnosis of any retinal disorders, including retinal detachment, retinal pigment epithelial detachment, serous retinopathy, or retinal vein occlusion.

### Screening and treatment procedures

Trial prescreening was allowed for patients undergoing first- or second-line treatment for advanced TNBC. H-scores for CDK6 and p-ERK were calculated for the tumor sample of each screened patient; the most recent tumor sample was used for this purpose. In order to avoid batch effects due to variables such as incubation time, minute variations in primary and secondary antibody concentrations, or different sample handling/fixing/storage protocols used in different hospitals, H-scores were normalized with *Z*-score to a reference set, consisting of 50 samples of primary TNBCs from four different hospitals mounted on a tissue microarray (TMA), restained along the patient’s sample each time. IHC staining was performed simultaneously on 2.5-μm TMA sections and patient samples of the study. IHC was performed using an automated protocol developed for the Autostainer Link automated slide staining system (Agilent Technologies, RRID: SCR_026889). All steps were performed on this staining platform using validated reagents, including deparaffinization, antigen retrieval (cell conditioning), and antibody incubation and detection. The following antibodies were used for IHC: phospho-p44/42 MAPK (Erk1/2; Thr202/Tyr204; Cell Signaling Technology, cat. # 9109, RRID: AB_2297442) and CDK6 (clone 98D/H8, Monoclonal Antibodies Core Unit, CNIO #AM98D). TMA and tumor stainings were acquired and digitalized using the ZEISS Axio Scan.Z1 Slide Scanner (RRID: SCR_020927). Digitalized images were analyzed with the Zeiss ZEN 3.2 lite software. H-scores were calculated by the following formula: [(% of area high intensity × 3) + (% of area medium intensity × 2) + (% of area low intensity × 1)]/100. CDK6 and p-ERK H-scores were calculated for the 50 samples and each new sample each time, so that each new sample could be referenced to a “standard” TNBC sample set. *Z*-score was calculated with the formula Z= (x-u) / (d/n^0.5^), in which x is the chosen datapoint, u is the mean value, and d is the SD of the distribution. Patients in which the *Z*-score for p-ERK, CDK6, or both was above the median were considered “positive” and candidates for trial inclusion.

**Figure 1.**
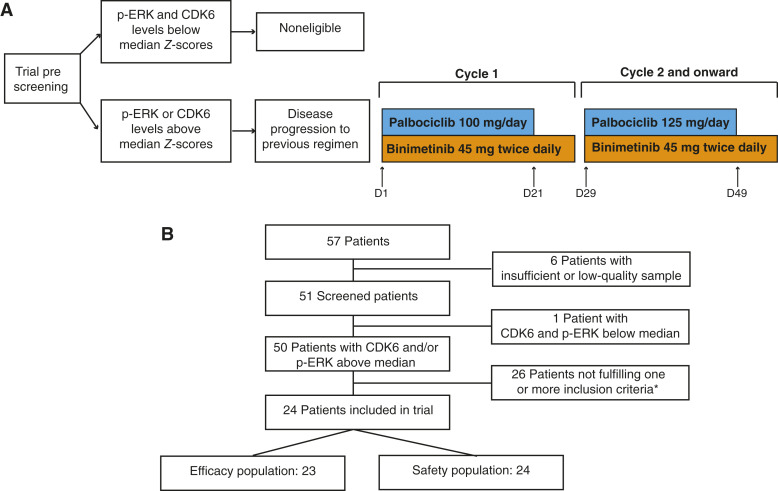
Trial schedule and CONSORT diagram. **A,** Basic trial schedule. Prescreened patients positive for either p-ERK and/or CDK6 were eligible after experiencing RECIST v1.1 disease progression to their first or second treatment line for advanced disease. Treatment consisted of continuous binimetinib (45 mg orally twice a day) and palbociclib (100 mg/day, orally) from days 1 to 21 of 28-day cycles. Palbociclib was allowed to be escalated to 125 mg/day in case of patients showing acceptable tolerance to 100/day during cycle 1. Treatment continued until disease progression, unacceptable toxicity, or investigator decision. D, day. **B,** CONSORT diagram. * The most frequent reasons for not meeting inclusion criteria were having received less than one or more than two treatment lines for advanced TNBC (*n* = 9), not having experienced disease progression to the previous treatment line according to RECIST v1.1 (*n* = 9), and inadequate organ function (*n* = 2). Eval., evaluation.

Once patients experienced disease progression according to RECIST v1.1, inclusion criteria were checked, and those complying all criteria were offered trial inclusion. Patients signing informed consent were started on binimetinib (45 mg twice a day) plus palbociclib (100 mg daily, days 1–21) in 28-day cycles. In case side effects equal or less than tolerable grade 2 were registered, patients were allowed to escalate palbociclib to 125 mg, according to the investigator’s decision. In case nontolerable grade 2 events associated with binimetinib were registered, patients would resume at 30 mg twice a day upon recovery. Palbociclib dose reductions as a result of toxicity were managed according to usual care. No reductions were allowed below 30 mg twice a day and 75 mg daily of binimetinib and palbociclib, respectively. Figure 1A depicts the trial schedule.

### Next-generation sequencing

Paraffin from 2.5-μm-thick tumor sample sections (8–10) was removed with xylene, followed by ethanol washes, and then DNA extraction was performed using High Pure FFPE DNA Isolation Kit (Roche, cat. # 06650767001) following the manufacturer’s instructions. Briefly, deparaffinized tissue pellet is incubated with lysis buffer and proteinase K for 60 minutes at 56°C. After inactivation of enzyme at 90°C, tissue lysates were placed into High Pure filter tubes, previously adding DNA-binding buffer and isopropanol. After washing column with buffers I and II, DNA was eluted in a fresh 1.5-mL reaction tube. DNA was then quantified using Qubit 1X dsDNA HS Assay Kit (Thermo Fisher Scientific, cat. # Q33230) in the Qubit 4 Fluorometer (Thermo Fisher Scientific, cat. # Q33238). DNA integrity evaluation was performed using the Genomic DNA Assay kit (Revvity, cat. # CLS760685) on the LabChip GX Touch Nucleic Acid Analyzer (Revvity, cat. # CLS138162, RRID: SCR_026830). Libraries were prepared using the Illumina DNA Prep with Enrichment v.2.5 kit (Illumina, cat. # 20077595) as recommended by the manufacturer. A set of 12-plex prehybridization pools were created and included for their hybridization with the Twist Bioscience for Illumina Exome 2.5 Panel (Illumina, cat. # 20077595). Library pools were quantified using Qubit 1X dsDNA HS Assay Kit (Thermo Fisher Scientific, cat. # Q33238) following the manufacturer’s suggested protocol in the Qubit 4 Fluorometer (Thermo Fisher Scientific, cat. # Q33238). The final library size were determined using High Sensitivity DNA Assay (Revvity, cat. # CLS760672) on the LabChip GX Touch (Revvity, cat. # CLS138162, RRID: SCR_026830). Libraries were sequenced using Illumina NovaSeq X Plus sequencing system (RRID: SCR_024568) at a loading concentration of 150 pmol/L in a 10B flow cell (2 × 150 bp). Illumina BCL Convert software were used for generating fastq files.

Variant calls were generated from the raw data using the Varca pipeline (18) in tumor-only mode, which implements the GATK’s best practices (19). Putative variants were then filtered to attempt to retain only somatic variants, keeping only those passing the pipeline quality control filters, with a frequency lower than 1% in the general population [gnomAD (20) and 1000 Genomes Project (21)] affecting primary transcripts (APPRIS = 1) and labeled as moderate- or high-impact variants by Ensembl’s VEP (22). Additionally, we discarded all variants with a variant allele frequency lower than 10%, as well as those annotated as intronic or classified in ClinVar as benign or likely benign. The remaining variants were used to estimate the mutational load per gene and per sample by counting the number of mutations regardless of zygosity.

### Sample size and statistical analysis

The trial was originally designed to determine the response rate of the combination. To this end, a Simon’s minimum–maximum design was adopted in order to detect a response rate of 30% or higher while minimizing the number of patients unnecessarily exposed to the treatment combination in case it was ineffective (stage 1: at least one response observed among 15 accrued patients was required to proceed to stage 2; 10 more patients would be accrued in this stage). These parameters were set before the publication of the phase III trial comparing sacituzumab govitecan against physicians’ chemotherapy choice in advanced TNBC, providing with high-quality evidence about the true efficacy rate of cytotoxic monotherapy regimens in this setting (2). Gemcitabine, capecitabine, eribulin, or vinorelbine were associated with a 5% response rate. Given that both study drugs were expected to display cytostatic activity, the investigators deemed the primary objective unreliable and unlikely to capture the activity of the combination. Thus, a protocol amendment was prepared and submitted to the authority, requesting the accrual of the 25 initially planned patients. Because the reported median progression-free survival (PFS) time was 1.7 months for the standard cytotoxic agents, the primary objective was set to demonstrate a 50% improvement in PFS (2.5 months) with this sample size, a beta error of 0.8, and an alpha error of 0.1. Secondary objectives were to determine safety, response rate, and biomarkers of activity of the combination. The efficacy population was defined as all patients included in the study that received at least one dose of study treatment and had at least one evaluation visit after the first dose. The safety population consisted of all patients included in the study who received at least one dose of the study regimen.

PFS was analyzed with the Kaplan–Meier method. Correlations between PFS time and ERK or CDK6 levels were analyzed with the Spearman coefficient. The percentage of patients with deleterious mutations among homologous recombination deficiency (HRD)-related genes experiencing prolonged or short disease control was compared with a Fisher test. Mutational loads between the two outcome groups (PFS>4 months vs. <4 months) were compared using a two-tailed *T* test. Genes were then ranked based on their T-statistics to identify those most strongly associated with each group.

## Results

### Study population and patient disposition

From December 2020 to June 2023, 51 patients were prescreened, of whom 50 were positive for CDK6 and/or p-ERK (Supplementary Fig. S1A). Examples of positive and negative screenings according to CDK6 and p-ERK are shown in Supplementary Fig. S1B. The first patient started treatment on January 22nd, 2021, and the last patient started treatment on July 12th, 2022. Of the 50 CDK6- and/or p-ERK–positive patients, 26 did not fulfil one or more inclusion criteria (Fig. 1). Due to competitive clinical trials and introduction of new reimbursed standards of care such as sacituzumab govitecan or trastuzumab deruxtecan, no further patients were started on trial despite positive screening tests from July 2022 to June 2023. At this point, 24 patients had been included in the trial; thus, with only one patient left to complete the planned accrual, the sponsor decided to close the study. The baseline clinical and demographic characteristics are shown in Table 1. The baseline CDK6 and p-ERK status of the 24 included patients is shown in Supplementary Fig. S1A. The majority of patients had received two treatment lines (54.2%; *N* = 13), and in 54.2% of the patients (*N* = 13), at least one of them had included immune checkpoint inhibitors. The CONSORT diagram is shown in Fig. 1B.

**Table 1.** Baseline characteristics.

Characteristic	*N* = 24 patients
Age (median, range)	53.8 (33.0–79.0)
Eastern Cooperative Oncology Group performance status	​
0	13 (54.2%)
1	11 (45.8%)
Time from diagnosis of advanced/metastatic disease (median, range) in months	16.5 (3.2–49.6)
Previous treatments for advanced disease	​
One line	11 (45.8%)
Two lines	13 (54.2%)
Received chemotherapy	24 (100%)
Received immunotherapy	13 (54.2%)

### Treatment and safety

Four (16.6%) patients escalated to palbociclib 125 mg twice a day in cycle 2, whereas the remaining continued on 100 mg twice a day. Patients received an average of 2.9 cycles (range, 1–17 cycles). The total number of cycles was 66. Six patients (25.0%) required one binimetinib reduction, whereas one (4.2%) patient required two. Two patients (8.3%) required one dose reduction of palbociclib.

Overall, toxicity was considerable. All patients (100%) experienced at least one adverse event; 17 patients required at least one binimetinib interruption [seven patients once (29.2%), eight patients twice (33.3%), one patient 4 times (4.2%), and one patient 14 times (4.2%)]. Fifteen patients required withholding palbociclib at some moment during their trial participation: seven patients once (29.2%), six patients twice (25%), and one and one patients 4 and 8 times (4.2%) each, respectively. The most frequent adverse events, occurring in at least 25% of the patients, were diarrhea (*n* = 17 patients; 70.8%), asthenia (70.8%), neutropenia (12 patients; 50%), skin rash (10 patients; 41.7%), anemia (nine patients; 37.5%), pyrexia and dyspnea (seven patients each; 29.2%), and thrombocytopenia (six patients; 25%). The treatment-emergent adverse events by preferred term Common Terminology Criteria for Adverse Events grade 2 or higher attributed to palbociclib or binimetinib occurring to at least 5% of the patients are displayed in Table 2. Although generally infrequent each, several different adverse events affected vision: grade 2 central vision loss, disruption of the photoreceptor inner segment–outer segment, retinopathy, or “ocular toxicity” (1 patient each – 4.2% – except from retinopathy – 2 patients) and grade 1 blindness, dry eye, dyschromatopsia, eyelid edema, macular scar, ocular toxicity, and photopsia (one patient each) and scintillating scotoma and vision blurred (2 patients – 8.3% – each), all attributed to binimetinib. There was as well one grade 3 scintillating scotoma event, also attributed to binimetinib. Regarding severe adverse events, there was one grade 3 mucositis event related to both study agents. Three more severe adverse events (pneumonitis, grade 3 pleural effusion, and grade 2 radicular compression; one patient each) were registered but related to disease progression in all three cases.

**Table 2.** Adverse events grade 2 or higher attributed to palbociclib and binimetinib (>5% of the patients).

Adverse Event	Palbociclib			Binimetinib	
	Grade 2	Grade 3	Grade 4	Grade 2	Grade 3
Neutropenia	4 (16.7%)	4 (16.7%)	2 (8.3%)	2 (8.3%)	1 (4.2%)
Anemia	4 (16.7%)	N/A	N/A	1 (4.2%)	N/A
Thrombocytopenia	3 (12.5%)	1 (4.2%)	N/A	N/A	1 (4.2%)
Diarrhea	4 (16.7%)	N/A	N/A	9 (37.5%)	2 (8.3%)
Mucositis	2 (8.3%)	1 (4.2%)	N/A	2 (8.3%)	1 (4.2%)
Creatine phosphokinase elevation	1 (4.2%)	N/A	N/A	3 (12.5%)	1 (4.2%)
Ejection fraction decrease	1 (4.2%)	N/A	N/A	2 (8.3%)	N/A
Rash	N/A	N/A	N/A	4 (16.7%)	1 (4.2%)
Retinopathy	N/A	N/A	N/A	2 (8.3%)	N/A
Asthenia	1 (4.2%)	1 (4.2%)	N/A	1 (4.2%)	1 (4.2%)

### Efficacy

**Figure 2.**
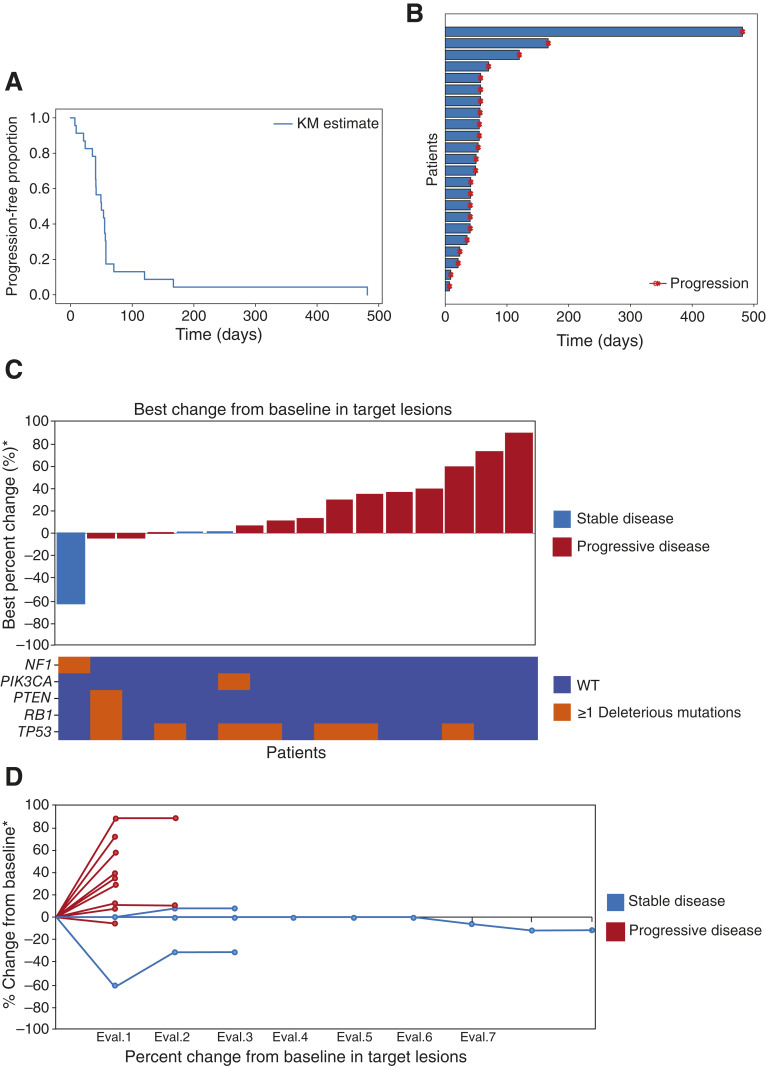
Efficacy results. **A,** Kaplan–Meier (KM) curve and (**B**) swimmer plot displaying PFS time for the efficacy population (*N* = 23). **C,** Waterfall plot showing the best overall response for the 16 patients with measurable disease. The mutational status of the most frequently mutated genes in TNBC is shown underneath the waterfall plot. **D,** Spider plot of the efficacy population.

Twenty-four patients initiated treatment, but one withdrew consent on day 12 of cycle 2 without undergoing a RECIST v1.1 assessment or experiencing clinical disease progression. Among the 23 patients included in the efficacy population, four experienced rapid clinical progression before the first scheduled CT scan at week 8 (after 9, 21, 41, and 50 days, respectively). The median PFS was 50 days (range, 9–482), as shown in the Kaplan–Meier curve (Fig. 2A); thus, the primary objective was not met. Considering that the median PFS on physician’s choice chemotherapy is 1.7 months (23), the swimmer plot (Fig. 2B) reveals a bimodal pattern of treatment duration: whereas most patients progressed within a similar timeframe, three patients (13.0%) achieved prolonged disease control, surpassing the 4-month threshold. These three outliers harbored convergent mutational landscapes, as further described below.

Among the 16 patients with measurable disease, the best response per RECIST v1.1 was stable disease in three (13.0%), whereas the remaining patients had progressive disease as their best response. The waterfall plot in Fig. 2C illustrates the maximum percentage change in target lesions from baseline. The spider plot (Fig. 2D) illustrates the individual trajectories of target lesion burden over time. Three patients exhibited an early and sustained stabilization of disease, whereas the majority experienced rapid progression within the first two assessments. Thus, despite rigorous biomarker-based selection, the combination did not demonstrate meaningful clinical activity in this molecularly enriched TNBC population.

### Correlative studies

Although the combination did not seem to have efficacy in the broad, heterogeneous TNBC patient population, the apparent binomial distribution in PFS time (with three patients experiencing a PFS >4 months) prompted us to investigate potential biomarkers of activity.

**Figure 3.**
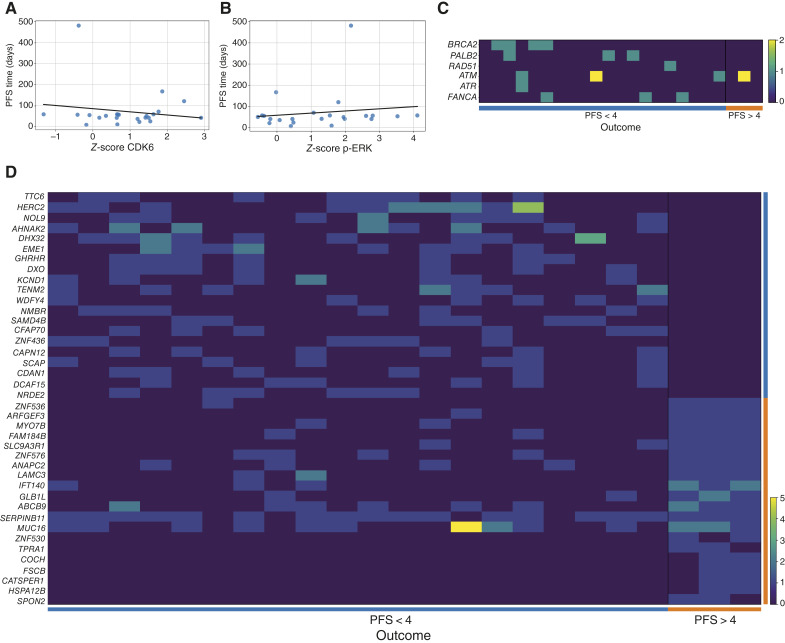
Correlative studies. **A,** Dot plot showing the correlation between CDK6 and (**B**) p-ERK staining (*Z*-score) and PFS time. The Pearson coefficients were −0.158 (*P* = 0.472) and 0.143 (*P* = 0.514). **C,** Mutational status of HR genes among patients with short (<4 months) or prolonged (>4 months) progression-free interval. Dark color indicates WT status of both alleles; green indicates a heterozygous deleterious mutation, and yellow indicates deleterious alterations in both alleles. **D,** Heatmap depicting the genes with differential mutational pattern between patients with short or long PFS, listed by their *T* test score. Darker colors indicate none or lower number of deleterious mutations across their exome, whereas lighter colors indicate increased number of deleterious mutations (up to 5).

First, we investigated the correlation between CDK6 and p-ERK staining intensity levels in baseline tumor samples and PFS, as in our initial study we found that the tumor models with higher activity of those signaling nodes were the most sensitive to the combination. The results, however, do not suggest any statistically significant or clinically meaningful relationship between CDK6 and/or p-ERK *Z*-scores and PFS time (Fig. 3A and B). These data suggest that whereas CDK6 and p-ERK hyperactivation may identify patients at higher risk of recurrence after a primary TNBC, they do not represent predictive biomarkers of benefit to combined CDK4/6 and MEK inhibition in advanced disease.

Next, we analyzed the WES data. The whole efficacy population was sequenced. When analyzing the distribution of the most frequently mutated genes in TNBC (*TP53*, *PIK3CA*, *PTEN*, *BRCA1*, *RB1*, *NF1*, *MYC*, *KMT2C*, *CDKN2A*, and *NOTCH*), no clear association was observed between mutational status and best response by RECIST v1.1. Responders and nonresponders displayed a heterogeneous pattern of alterations, suggesting that the clinical benefit observed in a small subset of patients is unlikely to be explained by mutations in these canonical TNBC-related genes alone (Fig. 2C).

Given the established relevance of HRD in TNBC and its association with increased sensitivity to platinum agents, PARP inhibitors, or immunotherapies, we also examined whether pathogenic mutations in canonical HRD genes (*BRCA1/2*, *PALB2*, *RAD51*, *BARD1*, *ATM*, *ATR*, *CHK1/2*, *FANCA*, and *FANCD2*) were enriched in patients who experienced prolonged disease control. Whereas no direct mechanistic link has been established between HRD and sensitivity to CDK4/6 or MEK inhibitors, we reasoned that tumors with impaired HR might rely more heavily on cell-cycle checkpoint signaling for survival. Moreover, MAPK inhibition can modulate lymphocyte infiltration in the tumor microenvironment, and HRD tumors may exhibit distinct immunologic features that could potentially enhance response to binimetinib. The results are shown in Fig. 3C. No patient harbored mutations in BRCA1, BARD1, CHK1/2, or FANCD2. Despite a suggestive visual pattern, a Fisher test indicated no statistically significant enrichment of HRD mutations in either outcome group (PFS >4 vs. <4 months).

To explore whether patterns of genomic alterations could distinguish patients who benefited from the combination, we constructed a heatmap including the top 20 most variably mutated genes [e.g., mutated in one category of patients and wild-type (WT) in the other, or at least more frequently in one subgroup vs. the other) across the cohort, ranked by a *T* test. Interestingly, patients with prolonged disease control (PFS >4 months) displayed a markedly distinct mutational profile (Fig. 3D). Several genes (e.g., *HERC2*, *AHNAK2*, and *TENM2*) were consistently WT in all three long-term responders but frequently mutated in others, whereas a different set of genes (e.g., *RPRD1A*, *MAP4K5*, and *ABCA1*) were mutated exclusively in long-term responders. Although limited by sample size, this finding suggests that mutational signatures might capture biological traits associated with differential sensitivity to the combination.

## Discussion

Advanced TNBC remains a highly complex and heterogeneous disease, with the unfortunate commonality of being almost uniformly lethal. Despite recent advances, the median OS for patients with metastatic TNBC remains below 24 months (1). The therapeutic landscape has evolved substantially with the introduction of antibody–drug conjugates and immune checkpoint inhibitors in early treatment lines, following demonstrated superiority over previous standards of care in phase III trials (1–3). However, these therapies are associated with significant toxicity and require intravenous administration (7–9). Upon progression after first- or second-line therapy, standard single-agent chemotherapies—such as capecitabine, vinorelbine, gemcitabine, or eribulin—remain the only available standard options, yet they yield a median PFS of less than 2 months (2). In this context, orally available, low-toxicity regimens would be highly desirable. Apart from PARP inhibitors for patients with germline BRCA1/2 mutations, no such alternatives currently exist for the broader advanced TNBC population. Against this therapeutic backdrop, identifying biologically driven vulnerabilities in TNBC—particularly those amenable to oral, targeted agents—remains a critical unmet need.

Our prior research demonstrated that hyperactivation of CDK6 and ERK in primary TNBC tumors was strongly associated with poor clinical outcomes, conferring a >9-fold increased risk of distant relapse (13). Given that both signaling nodes are pharmacologically targetable, we explored their therapeutic relevance in preclinical models. In patient-derived xenografts and spontaneous murine TNBC models, dual inhibition with palbociclib and GDC0994 (an ERK inhibitor) led to marked tumor shrinkage and significantly extended OS. These findings aligned with independent reports showing synergistic antitumor activity between MAPK pathway inhibitors and CDK4/6 inhibitors across multiple tumor types (14, 24, 25). Encouraged by these data, we designed a clinical trial to test the combination of palbociclib and binimetinib—targeting CDK6 and MEK, respectively—in patients with advanced TNBC, aiming for including patients enriched for baseline hyperactivation of CDK6 and/or ERK.

Real-time assessment of kinase activity in clinical settings remains impractical. Protein and phosphoprotein quantification from formalin-fixed, paraffin-embedded samples is technically demanding and not readily scalable across multicentric trials. Moreover, variability in analytic platforms and the lack of standardized pipelines closely mirror the initial challenges faced during the early implementation of next-generation sequencing in precision oncology trials. To circumvent these limitations, we opted for IHC quantification of CDK6 and p-ERK using standardized staining and normalization to a reference TNBC TMA. This approach was supported by prior observations linking staining intensity to kinase activity for selected targets (13). The fact that 98% of screened patients met the predefined positivity threshold reinforces the notion that elevated CDK6 and/or p-ERK levels are strongly enriched in the metastatic TNBC population—despite being present in only ∼25% of early TNBC cases—thus validating their prognostic relevance.

Unfortunately, despite the clear prognostic role of CDK6 and p-ERK hyperactivation, our findings do not support their value as predictive biomarkers of benefit to this therapeutic combination. The observed median PFS of less than 2 months, coupled with frequent treatment interruptions, resembles the clinical performance of standard cytotoxic agents—rendering this regimen suitable, at best, for late-stage disease with limited therapeutic alternatives. Objective responses were rare, with only one patient achieving a RECIST v1.1 response and just 13% of patients experiencing prolonged disease stabilization.

Moreover, this modest efficacy came at a nonnegligible toxicity cost. Hematologic toxicity—particularly neutropenia—frequently required treatment delays or dose adjustments. Additionally, symptomatic adverse events such as fatigue and diarrhea were common and clinically meaningful, especially in this palliative context. Of particular concern was the high incidence of ocular toxicity, which, although generally low-grade, included several distinct manifestations and is aligned with the class-effect of MEK inhibition (26, 27). In the palliative setting of advanced TNBC, visual disturbances may carry an especially disproportionate psychologic and functional impact. Unlike other adverse events such as fatigue or diarrhea, which are familiar and often transient, even mild visual symptoms can be deeply alarming. Vision loss strikes at the core of patient autonomy and quality of life, and it is uniquely distressing in a context in which time is limited and adaptation is no longer feasible.

These results also contribute to the debate about the utility of CDK4/6 inhibition in TNBC. Whereas CDK4/6 inhibitors have revolutionized the treatment of hormone receptor–positive disease (28–30), their role in TNBC has remained uncertain and largely unsupported by clinical evidence, with the exception of one clinical trial that combined the intravenous inhibitor trilaciclib with chemotherapy (31). Preclinical models, including our own, suggested that CDK6 hyperactivation might confer vulnerability to CDK4/6-targeting strategies, particularly when combined with MAPK pathway inhibition (13–17). The seminal work that prompted the study of palbociclib in hormone receptor–positive breast cancer also suggested activity in several TNBC preclinical models, although of lower magnitude (32). However, the clinical translation of this hypothesis has proven elusive (33–35). Our study—conducted in a molecularly selected population with CDK6 and/or p-ERK hyperactivation—did not demonstrate meaningful efficacy, even under highly controlled and biologically rational conditions. Taken together with the observed toxicity, these findings argue against the clinical viability of this combination in TNBC, and more broadly, challenge the notion that CDK4/6 inhibition is a suitable therapeutic avenue for this disease subtype. Although it is possible that our biomarker threshold was too permissive—based on median *Z*-scores rather than upper quartile cutoffs as in previous preclinical work—the absence of correlation between biomarker levels and clinical outcome (Fig. 3A and B) further undermines the case for refinement rather than rejection of this approach.

Genomic analyses, especially when coupled with computational clustering strategies, now enable a deeper understanding of outlier clinical behavior. In this study, we performed WES on all patients in the efficacy population to explore whether specific mutational patterns could help explain the observed dichotomic PFS pattern. When examining mutations in canonical TNBC drivers, no clear association with response was observed (Fig. 2C). Responders and nonresponders exhibited a heterogeneous mix of alterations. The status of HRD might explain longer responses, given its known role in sensitizing TNBC tumors to certain agents. Although HRD is not mechanistically linked to MEK or CDK4/6 inhibition yet, we hypothesized that HRD tumors might rely more heavily on compensatory pathways such as MAPK or CDK-mediated cell-cycle checkpoints and thus be indirectly sensitized. Additionally, MEK inhibition has been reported to modulate the tumor immune microenvironment (36, 37), and HRD tumors may harbor distinct immunologic profiles. However, the results shown in Fig. 3C show a clear lack of association between mutations in HR genes and PFS time.

Intriguingly, the analysis of the most differentially mutated genes across the cohort yielded a coherent separation between patients with poor and prolonged disease control (Fig. 3D). Long-term responders displayed a distinct mutational signature. Several genes were uniformly WT in these patients yet frequently mutated in others, in which a different subset of genes showed the opposite pattern. Although caution is warranted because the small sample size, these findings suggest that composite mutational patterns—not individual alterations—might capture biological traits underlying sensitivity to MEK and CDK4/6 inhibition. Although the functional dissection of these differences is beyond the scope of this study, it is worth noting that several of these genes are involved in biological processes potentially relevant to response. For example, HERC2 is a large ubiquitin ligase involved in DNA damage response (DDR), particularly in HR. It acts as a scaffold for the recruitment and stabilization of proteins such as BRCA1 or 53BP1 at sites of DNA double-strand breaks (38). HERC2 was heavily mutated in nonresponders. Whereas causality cannot be established, several hypotheses make this association plausible: for example, palbociclib requires intact cell-cycle checkpoints; an impaired DDR might accumulate excessive DNA damage without effectively arresting, leading to resistance. In addition, palbociclib can induce senescence rather than apoptosis (39), a process that may be more durable in the presence of functional DDR. *AHNAK2*, another gene conserved in the patients with PFS>4 months, has been implicated in EMT and cell migration (40); its preservation in responders might reflect a more epithelial, drug-sensitive phenotype. Finally, emerging data point toward *TENM2* as a mediator of cell–cell interactions (41); mutations in this gene might disrupt cellular architecture and promote metastatic shedding. Although more speculative, it is worth noting that several of the genes preferentially mutated in long-term responders, such as *COCH* or *SPON2*, are involved in innate immune system modulation (42) and M2-polarized tumor-associated macrophage infiltration, suggesting that a disfunction in these genes might underlie clinical benefit in this subgroup.

Several preclinical studies have provided strong rationale for combining MAPK and CDK4/6 inhibitors. Whereas multiple clinical trials have been launched to explore this strategy, to our knowledge, this is the first reported study to test this combination. Despite a rational biomarker-driven design and robust preclinical evidence, the overall clinical efficacy observed in this trial was limited, with a median PFS comparable with that of conventional chemotherapy in this setting. Additionally, the regimen was associated with considerable toxicity, which poses a challenge for its further development. Nonetheless, the observed bimodal distribution of clinical outcomes—combined with distinct mutational profiles among long-term responders—highlights the potential of genomic stratification to uncover subpopulations that may derive benefit. These findings should be explored in independent cohorts and suggest that, if this combination is to be pursued, a genomically selected approach will be essential. Future mechanistic studies may help clarify the biological basis of response and resistance, ultimately informing better patient selection strategies for these targeted therapies in TNBC.

## Supplementary Material

Figure S1 Figure S1 shows CDK6 and p-ERK positivity in screened samples

Table S1 Representativeness of Study Participants table

## Data Availability

Whole-exome sequencing (WES) data presented in this study have been deposited on the public repository *The Sequence Read Archive* (NCBI SRA) with the following accession code: PRJNA1288724. All other data are in the main article, supplementary files, or upon request from the corresponding author.
