# Supplementary material for: A Phase IB Study of Binimetinib and Palbociclib in Molecularly Selected Advanced Triple-Negative Breast Cancer
Source: Cancer Res Commun. 2025 Sep 29;5(9):1728–37. doi: 10.1158/2767-9764.CRC-25-0428 (PMC12477833; doi:10.1158/2767-9764.CRC-25-0428)
Supplement: Figure S1 — shows CDK6 and p-ERK positivity in screened samples [file crc-25-0428_figure_s1_suppsf1.docx]

**Supplementary Data**

**Supplementary figure 1**

**
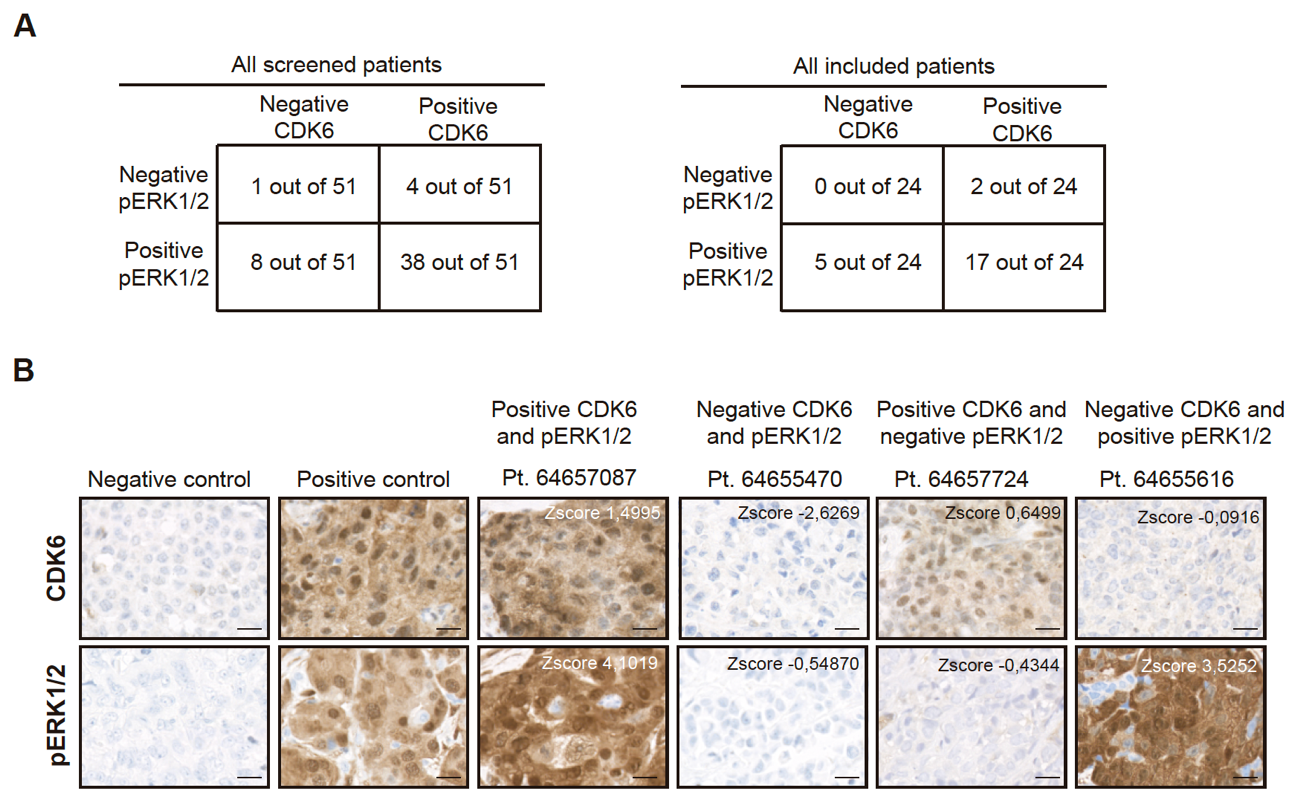
**

**Supplementary Figure 1: CDK6 and p-ERK positivity in screened samples. (A)** The 2x2 tables show the number of patients with H-scores of either of the two probes above or below the cut-off threshold, in the full screening population and in the trial population, respectively. **(B)** Examples of CDK6 and p-ERK staining in positive and negative controls, and in double-positive, single-positive and double-negative tumor samples. Scale bar 20μm
