# Supplementary material for: A Phase IB Study of Binimetinib and Palbociclib in Molecularly Selected Advanced Triple-Negative Breast Cancer
Source: Cancer Res Commun. 2025 Sep 29;5(9):1728–37. doi: 10.1158/2767-9764.CRC-25-0428 (PMC12477833; doi:10.1158/2767-9764.CRC-25-0428)
Supplement: Table S1 — Representativeness of Study Participants table [file crc-25-0428_table_s1_suppst1.docx]

**Table S1: Representativeness of Study Participants**

| Cancer type(s)/subtype(s)/stage(s)/condition | Triple negative breast cancer (TNBC) |
| --- | --- |
| Considerations related to: |  |
| Sex | TNBC, as all other subtypes of breast cancer, is a predominantly female disease and is rare in men. Male breast cancer represents less than 1% of all breast cancers diagnosed each year. In this study, no male TNBC patients were recruited. |
| Age | The globally median age at the time of all breast cancer diagnosis is around 60, while in TNBC it is closer to 54. |
| Race/ethnicity | All the patients enrolled in the study were Caucasian. |
| Geography | This study was conducted in Spain at three locations: Madrid, Valencia and Lerida. |
| Overall representativeness of this study | This phase I safety study was conducted in 24 female patients enrolled in Spain. The median age of patients in this study was 53.8 and the range was 33-79 years, which is representative of most patients with this cancer type. |
